# Supplementary material for: Small molecule stabilization of diverse amyloidogenic immunoglobulin light chains revealed by hydrogen-deuterium exchange mass spectrometry
Source: bioRxiv. 2026 Jan 8:2026.01.07.698275. Preprint. [Version 1] doi: 10.64898/2026.01.07.698275 (PMC12803214; doi:10.64898/2026.01.07.698275)
Supplement: 1 [file NIHPP2026.01.07.698275v1-supplement-1.pdf]

bioRxiv preprint doi: https://doi.org/10.64898/2026.01.07.698275; this version posted January 8, 2026. The copyright holder for this preprint (which was not certified by peer review) is the author/funder, who has granted bioRxiv a license to display the preprint in perpetuity. It is made available under aCC-BY 4.0 International license.

Small molecule stabilization of diverse amyloidogenic immunoglobulin light chains revealed by hydrogen-deuterium exchange mass spectrometry

Daniele Peterle, Nicholas L. Yan, Elena S. Klimtchuk, Thomas E. Wales, Olga Gursky, Jeffery W. Kelly, John R. Engen, Gareth J. Morgan

Supplementary Appendix

Protein sequences

Supplementary Figures S1-S8

bioRxiv preprint doi: https://doi.org/10.64898/2026.01.07.698275; this version posted January 8, 2026. The copyright holder for this preprint (which was not certified by peer review) is the author/funder, who has granted bioRxiv a license to display the preprint in perpetuity. It is made available under aCC-BY 4.0 International license.

Protein sequences

>AL
NFMLTQPHSVSESPGKTVTISCTRSSGSIASTYVQWYQQRPGSAPTNVIFEDNERPSGVPDRFSGSIDSSSNSAYLTISGLKTED
EADYYCQSYGTNNWVFGGGTKLTVL
GQPKAAPSVTLFPPSSEELQANKATLVCLISDFYPGAVTVAWKADSSPVKAGVETTTPSKQSNNKYAASSYLSLTPEQWKSHRSY
SCQVTHEGSTVEKTVAPTECS
>GL
NFMLTQPHSVSESPGKTVTISCTRSSGSIASNYVQWYQQRPGSSPTTVIYEDNQRPSGVPDRFSGSIDSSSNSASLTISGLKTED
EADYYCQSYDSSNWVFGGGTKLTVL
GQPKAAPSVTLFPPSSEELQANKATLVCLISDFYPGAVTVAWKADSSPVKAGVETTTPSKQSNNKYAASSYLSLTPEQWKSHRSY
SCQVTHEGSTVEKTVAPTECS
>MM
NFMLNQPHSVSESPGKTVTISCTRSSGNIDSNYVQWYQQRPGSAPITVIYEDNQRPSGVPDRFAGSIDRSSNSASLTISGLKTED
EADYYCQSYDARNVVFGGGTRLTVL
GQPKAAPSVTLFPPSSEELQANKATLVCLISDFYPGAVTVAWKADSSPVKAGVETTTPSKQSNNKYAASSYLSLTPEQWKSHKSY
SCQVTHEGSTVEKTVAPTECS
>H3
QSVLTQPPSTSGTPGQRVTISCSGSSSNIETNTVNWYQQLPGTAPKLVMHTNNQRPSGVPDRFSGSRSGTSASLAIGGLQSEDEA
DYFCAAWDDNLNGVIFGGGTKLTVL
GQPKAAPSVTLFPPSSEELQANKATLVCLISDFYPGAVTVAWKADSSPVKAGVETTTPSKQSNNKYAASSYLSLTPEQWKSHKSY
SCQVTHEGSTVEKTVAPTECS
>H6
QSVLTQPPSVSAAPGQKVTISCSGNNSNIGKNYVSWYQQLPGRTPKVIMYENNKRSSGIPDRFSGSKSGTSATLGITGLQTGDEA
DYYCGVWDSSLSGGVFGGGTKVTVL
GQPKAAPSVTLFPPSSEELQANKATLVCLISDFYPGAVTVAWKADSSPVKAGVETTTPSKQSNNKYAASSYLSLTPEQWKSHRSY
SCQVTHEGSTVEKTVAPTECS
>H7
QSVLTQPPSVSAAPGQKVTISCSNVGKNFVSWYQQFPGTAPKVVIYDTDKRPSDIPDRFSGSKSGTSATLDITGLQTGDEADYYC
GTWDSGLNGGVFGGGTKVTVL
GQPKAAPSVTLFPPSSEELQANKATLVCLISDFYPGAVTVAWKADSSPVKAGVETTTPSKQSNNKYAASSYLSLTPEQWKSHKSY
SCQVTHEGSTVEKTVAPTECS
>H9
QSALTQPPSASGSPGQSVTISCTGTSSDVGGSDSVSWYQQHPGKAPKLIIYEVSQRPSGVPNRFSGSKSGNTASLTVSGLQAEDD
ADYYCSSYGGDNNLFFGGGTKVTVL
GQPKAAPSVTLFPPSSEELQANKATLVCLISDFYPGAVTVAWKADSSPVKAGVETTTPSKQSNNKYAASSYLSLTPEQWKSHRSY
SCQVTHEGSTVEKTVAPTECS
>H16
QSALTQPASVSGSPGLSITISCTGTSSDIGGYNSVSWYQQHPGKAPKLIIYEVSNRPSGISNRFSGSKSGYTASLTISGLQAEDE
ADYYCSSYTNSGILFGGGTELTVL
GQPKAAPSVTLFPPSSEELQANKATLVCLISDFYPGAVTVAWKADSSPVKAGVETTTPSKQSNNKYAASSYLSLTPEQWKSHKSY
SCQVTHEGSTVEKTVAPTECS
>MCG
QSALTQPPSASGSLGQSVTISCTGTSSDVGGYNYVSWYQQHAGKAPKVIIYEVNKRPSGVPDRFSGSKSGNTASLTVSGLQAEDE
ADYYCSSYEGSDNFVFGTGTKVTVL
GQPKANPTVTLFPPSSEELQANKATLVCLISDFYPGAVTVAWKADGSPVKAGVETTKPSKQSNNKYAASSYLSLTPEQWKSHRSY
SCQVTHEGSTVEKTVAPTECS

bioRxiv preprint doi: https://doi.org/10.64898/2026.01.07.698275; this version posted January 8, 2026. The copyright holder for this preprint (which was not certified by peer review) is the author/funder, who has granted bioRxiv a license to display the preprint in perpetuity. It is made available under aCC-BY 4.0 International license.

Supplementary Figures

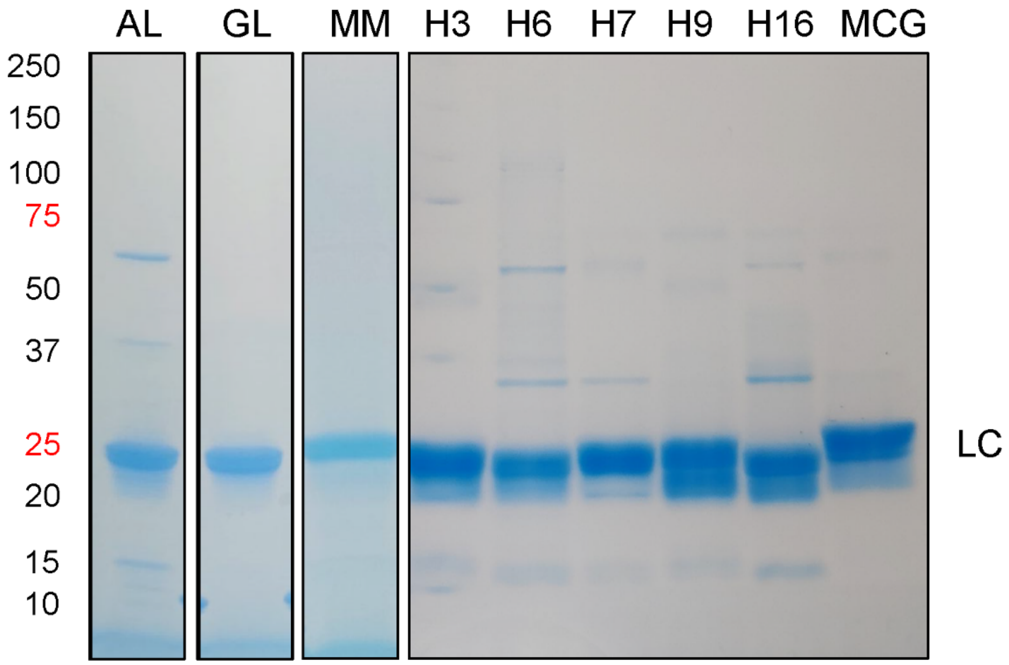

Figure S1. SDS-PAGE characterization of the nine full-length LCs used in this study. Proteins were run on a 10–20% acrylamide gel under reducing conditions and the gels were stained with Coomassie. 7 µg of each protein sample was loaded per lane. Molecular weight markers are indicated on the left. The secondary bands underneath the main LC band appear to be alternative conformers the main LC protein, since no truncated species were observed by mass spectrometry.

bioRxiv preprint doi: https://doi.org/10.64898/2026.01.07.698275; this version posted January 8, 2026. The copyright holder for this preprint (which was not certified by peer review) is the author/funder, who has granted bioRxiv a license to display the preprint in perpetuity. It is made available under aCC-BY 4.0 International license.

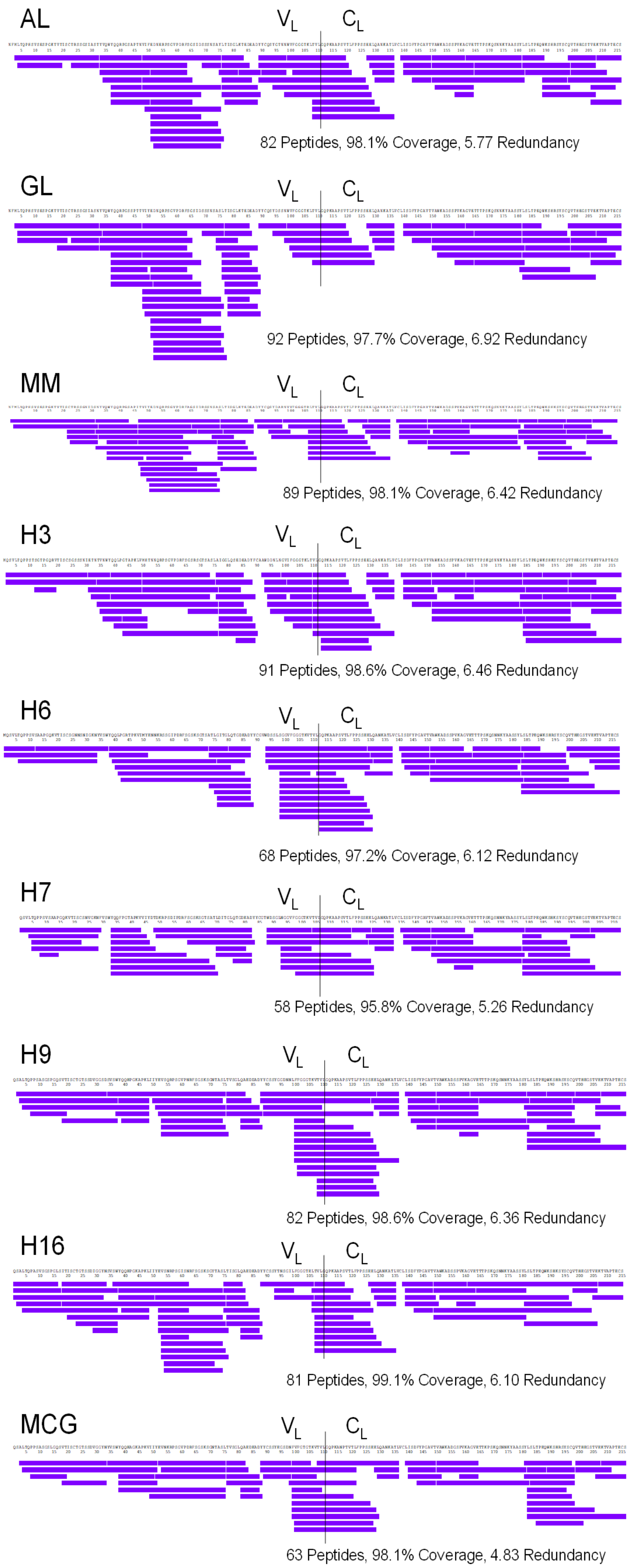

bioRxiv preprint doi: https://doi.org/10.64898/2026.01.07.698275; this version posted January 8, 2026. The copyright holder for this preprint (which was not certified by peer review) is the author/funder, who has granted bioRxiv a license to display the preprint in perpetuity. It is made available under aCC-BY 4.0 International license.

Figure S2. Sequence coverage maps of LCs constructs used in this study. Horizontal purple bars below LC sequence indicate the peptides for which HDX was followed. Vertical lines mark the separation between the V_L and the C_L. The number of peptides, sequence coverage (%), and average redundancy values are reported for each LC.

bioRxiv preprint doi: https://doi.org/10.64898/2026.01.07.698275; this version posted January 8, 2026. The copyright holder for this preprint (which was not certified by peer review) is the author/funder, who has granted bioRxiv a license to display the preprint in perpetuity. It is made available under aCC-BY 4.0 International license.

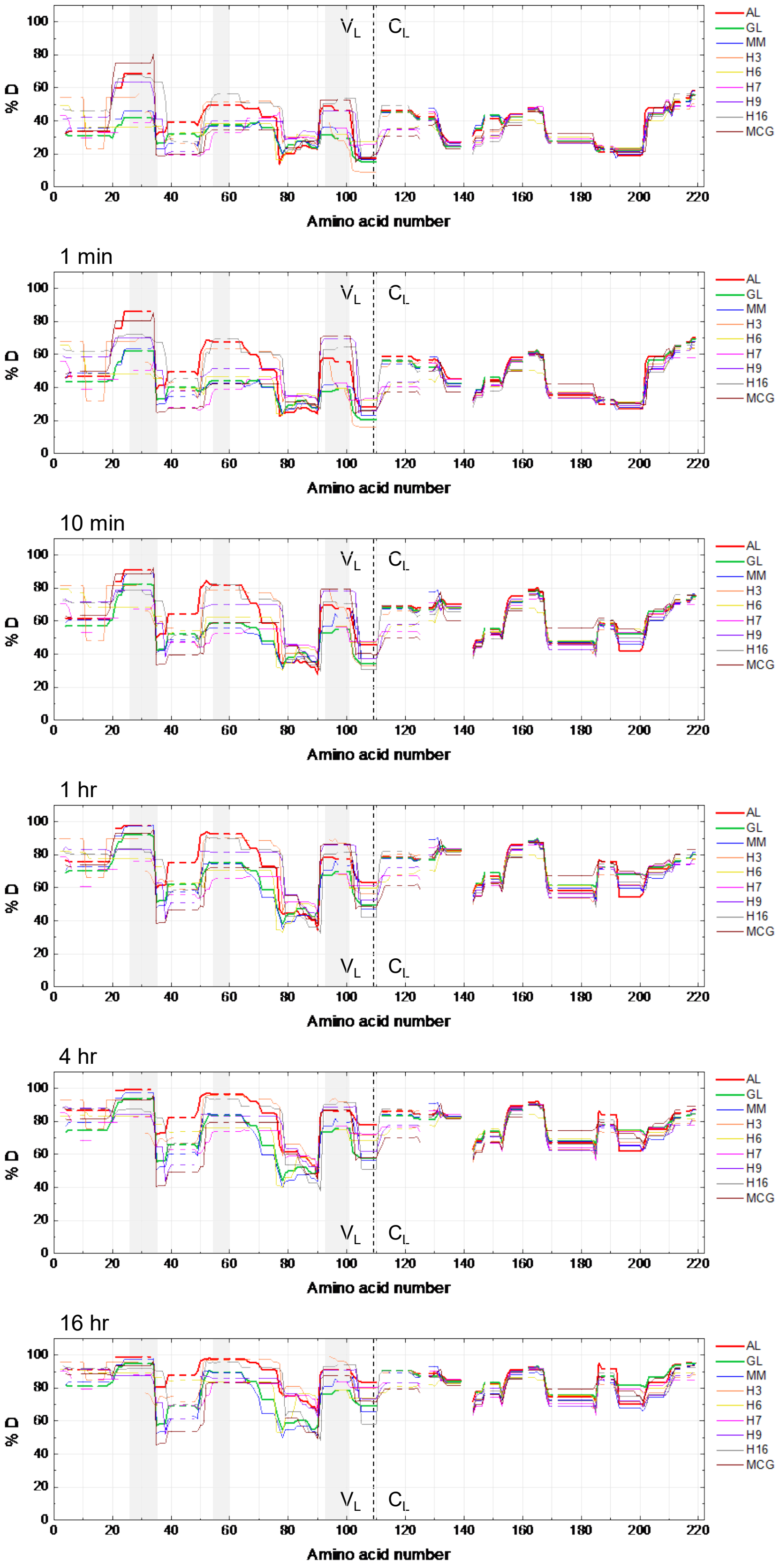

bioRxiv preprint doi: https://doi.org/10.64898/2026.01.07.698275; this version posted January 8, 2026. The copyright holder for this preprint (which was not certified by peer review) is the author/funder, who has granted bioRxiv a license to display the preprint in perpetuity. It is made available under aCC-BY 4.0 International license.

Figure S3. Single-residue aligned skyline plots for all light chains (LCs) at six different labeling time points: 10 seconds, 1 minute, 10 minutes, 1 hour, 4 hours, and 16 hours. Percent deuterium incorporation (%D) is plotted against amino acid number, with vertical dashed lines indicating the boundary between V_L and C_L domains. Gray boxes indicate the CDR regions. Each line represents a specific LC isoform, defined in the legend on the right.

bioRxiv preprint doi: https://doi.org/10.64898/2026.01.07.698275; this version posted January 8, 2026. The copyright holder for this preprint (which was not certified by peer review) is the author/funder, who has granted bioRxiv a license to display the preprint in perpetuity. It is made available under aCC-BY 4.0 International license.

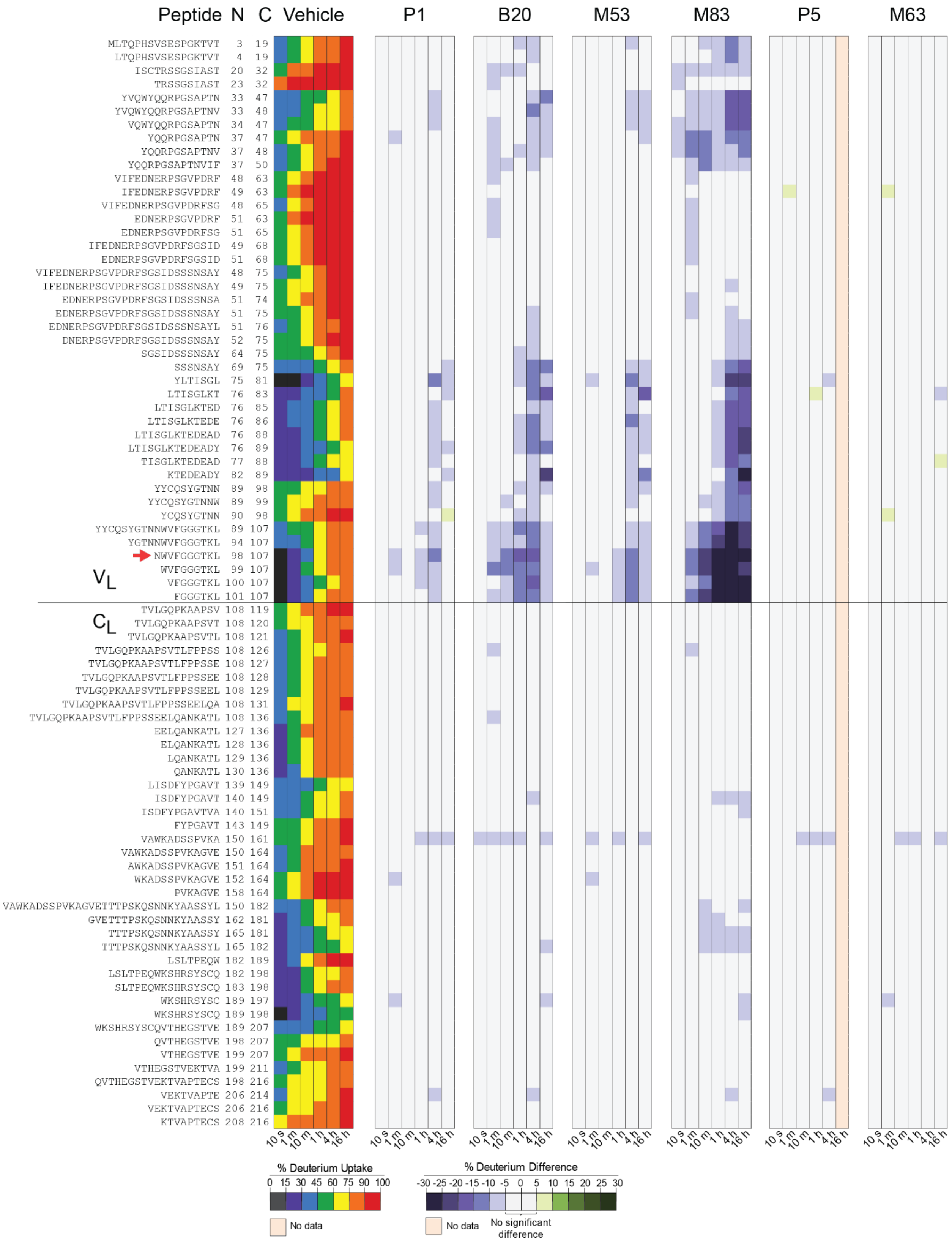

bioRxiv preprint doi: https://doi.org/10.64898/2026.01.07.698275; this version posted January 8, 2026. The copyright holder for this preprint (which was not certified by peer review) is the author/funder, who has granted bioRxiv a license to display the preprint in perpetuity. It is made available under aCC-BY 4.0 International license.

Figure S4. HDX-MS screening of six different kinetic stabilizers. Peptide chiclet plots display differences in the percent deuterium uptake across the sequence of AL LC upon binding of each compound. The compounds screened were P1, B20, M53, M83, P5, and M63. On the left, the absolute uptake of AL LC (no compound) serves as the baseline for comparison. M83 shows the most significant protection, followed by B20, M53, and P1, with decreasing levels of protection. P5 and M63 do not show notable differences in HDX. The horizontal line separates the variable (V_L) and constant (C_L) domains. Regions with no data (peach) or no change (gray) are indicated. The AL LC peptide ^{101}NWVFGGGTKL^{110} (corresponding to sequential positions 98-107) is highlighted with a red arrow.

bioRxiv preprint doi: https://doi.org/10.64898/2026.01.07.698275; this version posted January 8, 2026. The copyright holder for this preprint (which was not certified by peer review) is the author/funder, who has granted bioRxiv a license to display the preprint in perpetuity. It is made available under aCC-BY 4.0 International license.

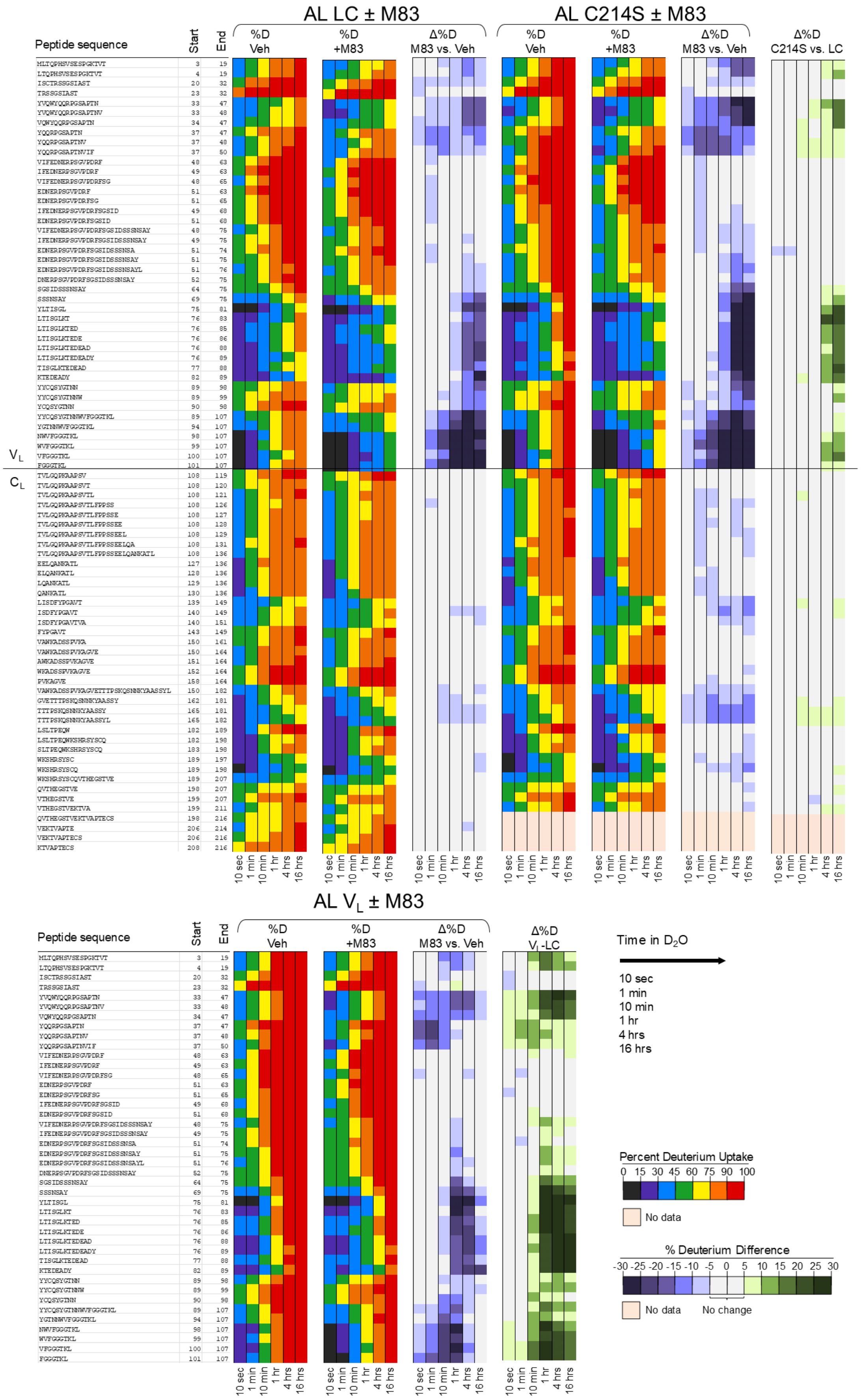

bioRxiv preprint doi: https://doi.org/10.64898/2026.01.07.698275; this version posted January 8, 2026. The copyright holder for this preprint (which was not certified by peer review) is the author/funder, who has granted bioRxiv a license to display the preprint in perpetuity. It is made available under aCC-BY 4.0 International license.

Figure S5. Binding of the kinetic stabilizer M83 to AL LC, C214S, and V_L monitored by HDX-MS. This figure presents the same data as Figure 3, displayed in a peptide-resolution chiclet plot format instead of residue-level heatmaps. Percent deuterium incorporation (%D) is shown for the protein in 0.15% DMSO vehicle and in the presence of M83, alongside the differences in deuterium uptake (Δ%D). Comparisons between constructs (C214S-LC and V_L-LC) are also shown. Horizontal lines separate V_L and C_L domains. Regions with no data (peach) or no change (gray) are indicated.

bioRxiv preprint doi: https://doi.org/10.64898/2026.01.07.698275; this version posted January 8, 2026. The copyright holder for this preprint (which was not certified by peer review) is the author/funder, who has granted bioRxiv a license to display the preprint in perpetuity. It is made available under aCC-BY 4.0 International license.

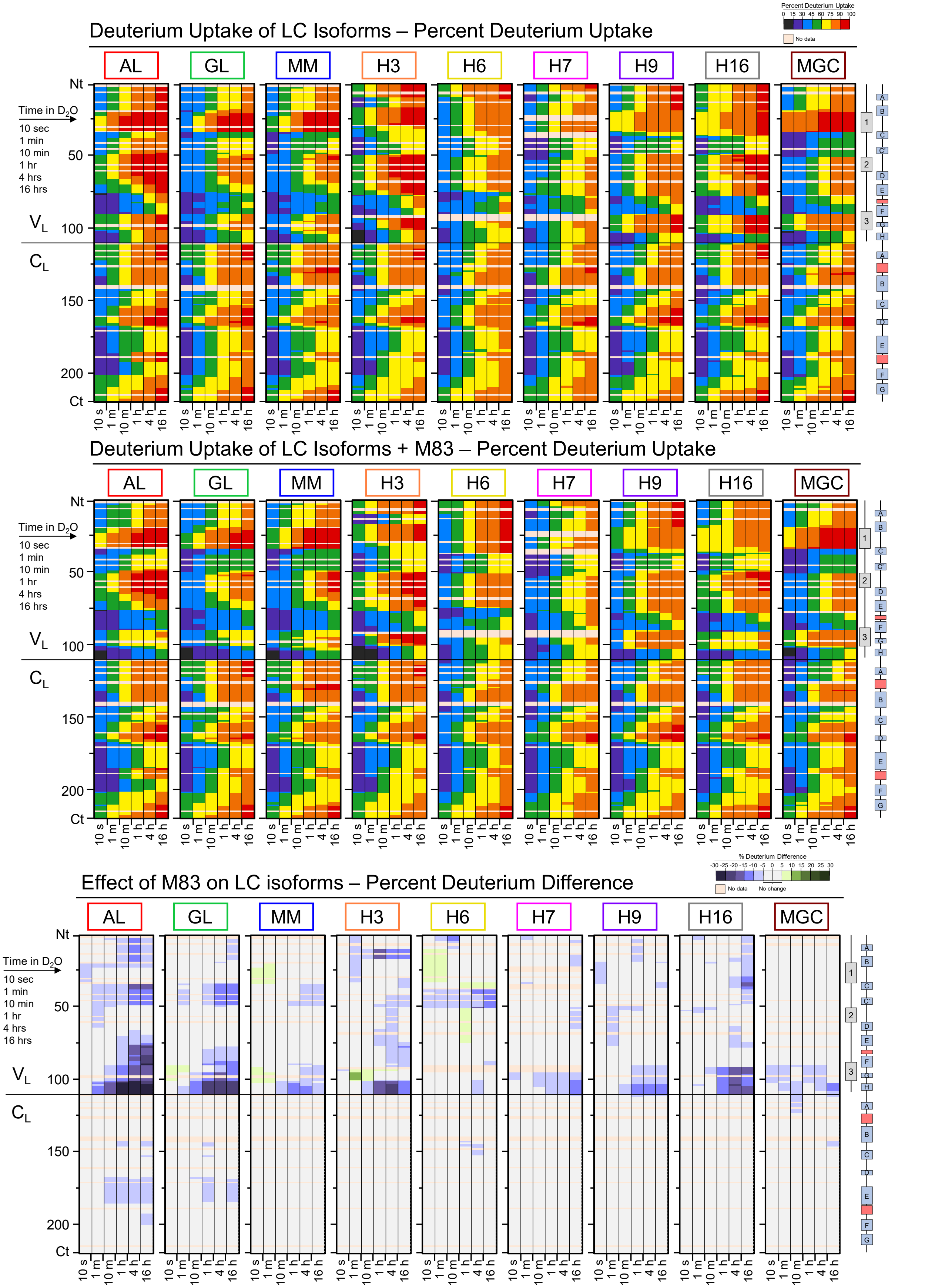

bioRxiv preprint doi: https://doi.org/10.64898/2026.01.07.698275; this version posted January 8, 2026. The copyright holder for this preprint (which was not certified by peer review) is the author/funder, who has granted bioRxiv a license to display the preprint in perpetuity. It is made available under aCC-BY 4.0 International license.

Figure S6. Single residue aligned heatmaps of LC isoforms with and without M83. Top panels: Percent deuterium uptake (%D) for all nine LC isoforms in the absence of M83. Middle panels: Percent deuterium uptake (%D) for LC isoforms in the presence of M83. Levels of deuterium uptake is color-coded as per legend, with red indicating high uptake (low protection) and blue indicating low uptake (high protection). Bottom Panels: Percent deuterium difference (Δ%D) plots showing the effect of M83 on deuterium uptake for each LC isoform. Blue regions indicate areas of increased protection upon M83 binding, while green regions represent areas with reduced protection. The solid horizontal line indicates the boundary between V_L and C_L domains.

bioRxiv preprint doi: https://doi.org/10.64898/2026.01.07.698275; this version posted January 8, 2026. The copyright holder for this preprint (which was not certified by peer review) is the author/funder, who has granted bioRxiv a license to display the preprint in perpetuity. It is made available under aCC-BY 4.0 International license.

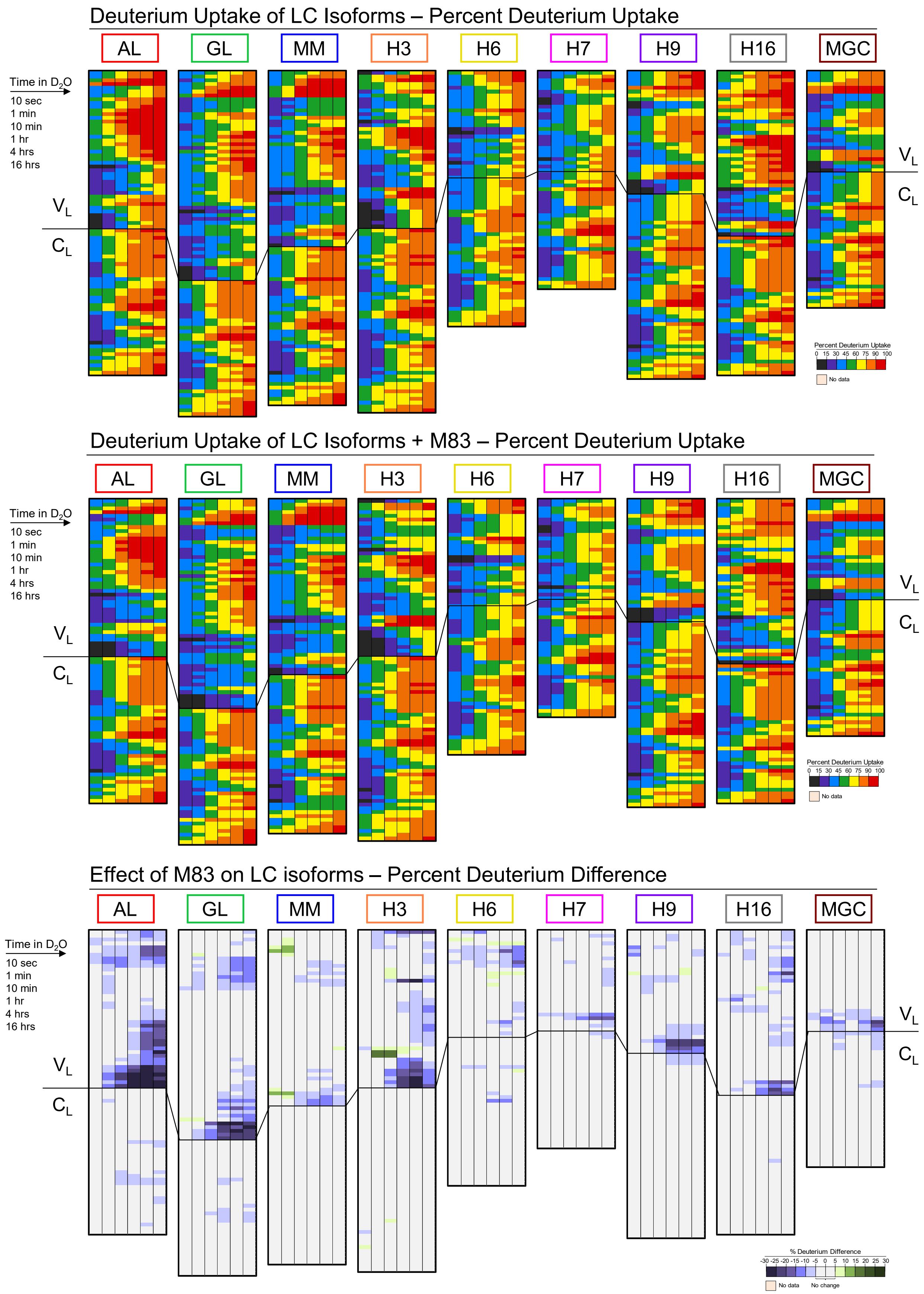

bioRxiv preprint doi: https://doi.org/10.64898/2026.01.07.698275; this version posted January 8, 2026. The copyright holder for this preprint (which was not certified by peer review) is the author/funder, who has granted bioRxiv a license to display the preprint in perpetuity. It is made available under aCC-BY 4.0 International license.

Figure S7. Peptide chiclet plots showing deuterium uptake of various LCs and the effect of M83. Top panel: Percent deuterium uptake (%D) for all nine LC isoforms in the absence of M83. Levels of deuterium uptake are color-coded as per the legend, with red indicating high uptake (low protection) and blue indicating low uptake (high protection). Middle panel: Percent deuterium uptake (%D) for all nine LC isoforms in the presence of M83. Bottom panel: Percent deuterium difference (Δ%D) plots showing the effect of M83 on deuterium uptake for each LC isoform. Blue regions indicate areas of increased protection upon M83 binding, while green regions represent areas with reduced protection. The solid horizontal line indicates the boundary between the variable domain (V_L) and the constant domain (C_L).

bioRxiv preprint doi: https://doi.org/10.64898/2026.01.07.698275; this version posted January 8, 2026. The copyright holder for this preprint (which was not certified by peer review) is the author/funder, who has granted bioRxiv a license to display the preprint in perpetuity. It is made available under aCC-BY 4.0 International license.

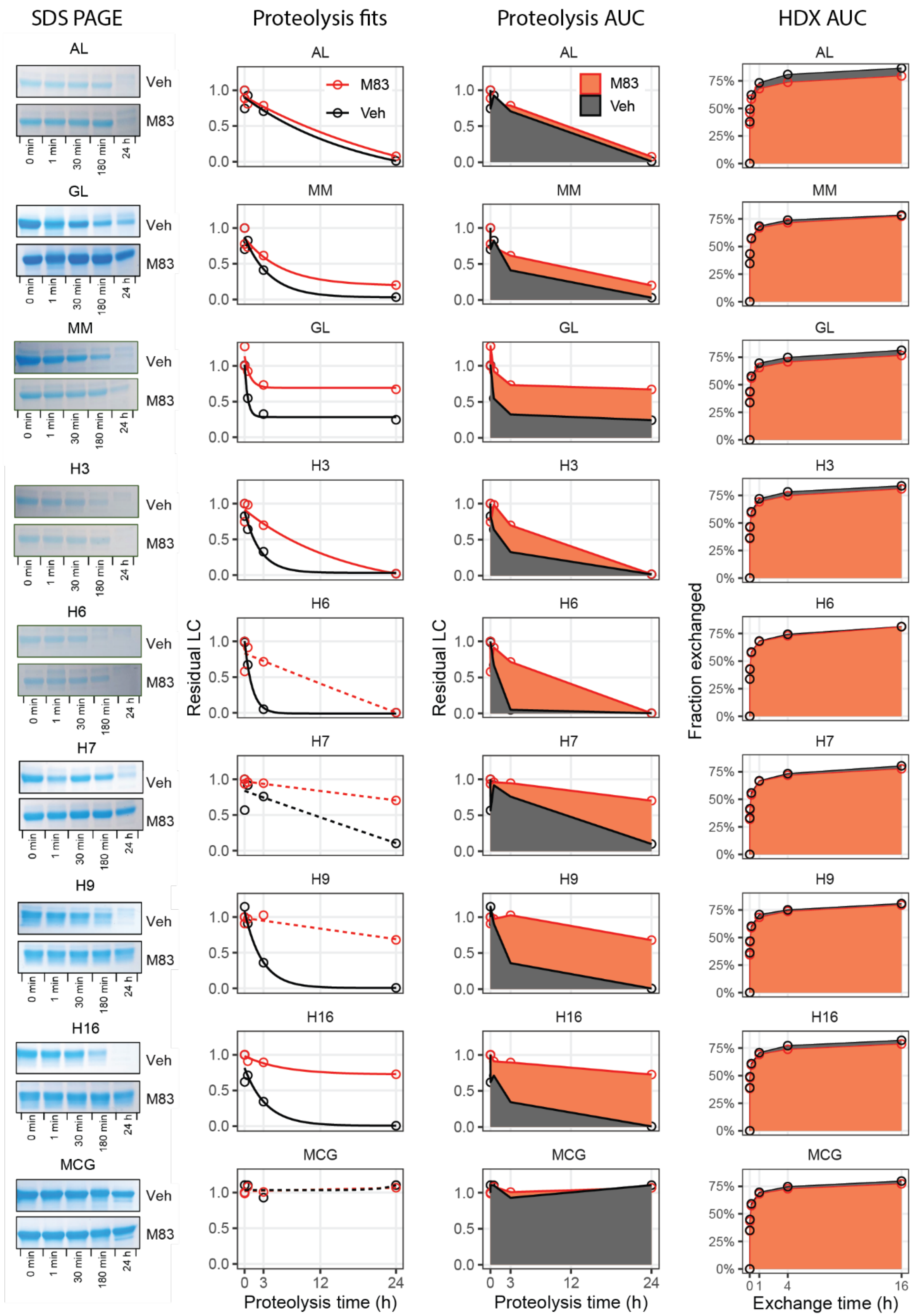

bioRxiv preprint doi: https://doi.org/10.64898/2026.01.07.698275; this version posted January 8, 2026. The copyright holder for this preprint (which was not certified by peer review) is the author/funder, who has granted bioRxiv a license to display the preprint in perpetuity. It is made available under aCC-BY 4.0 International license.

Figure S8. Limited proteolysis assay of FL LCs with and without M83 monitored by SDS-PAGE. FL LCs were tested at a concentration of 8.3 µM, with M83 at 50 µM (1:6 molar ratio) in 10 mM phosphate buffer, pH 7.4, 150 mM NaCl, at 37°C and 450 rpm. Proteolysis reaction was initiated by addition of trypsin at 0.232 µM. Time points (0, 1, 30, 180 minutes, and 24 hours) are shown for digestion experiments. "+" and "-" refer to digestion in the presence and absence of M83, respectively. Gels (10-20%) were run under reducing conditions and stained with Coomassie Blue. Alcohol dehydrogenase was included as a loading control (not shown). Data were fitted to a single exponential decay model and the area under the curve (AUC) was calculated. Dashed lines show data where the fits did not converge. For comparison, the average AUC for all residues was calculated for each FL LC.
